# Supplementary material for: Biomarkers of Neurological Damage: From Acute Stage to Post-Acute Sequelae of COVID-19
Source: Cells. 2023 Sep 13;12(18):2270. doi: 10.3390/cells12182270 (PMC10526816; doi:10.3390/cells12182270)
Supplement: Supplementary file 1 [file cells-12-02270-s001.zip › cells-2554670-supplementary.pdf]

Table S1. Demographic and clinical characteristics of 117 living COVID-19 patients stratified by post-COVID visit receiving.

	Post-COVID visit		
	**Accept (n=77)**	**Decline (n=40)**	**p value***
**Female, n (%)**	36 (46.8)	20 (50.0)	ns
**Age, median (IQR) (years)**	60 (52-71)	66 (57-70)	ns
**Severe COVID-19, n (%)**	34 (44.2)	25 (62.5)	ns
**Comorbidity**			
Any, n (%)	46 (59.7)	21 (52.5)	ns
Hypertension, n (%)	29 (36.7)	14 (35.0)	ns
Cardiovascular, n (%)	9 (11.7)	7 (17.5)	ns
Diabetes, n (%)	13 (16.9)	5 (12.5)	ns
Pulmonary, n (%)	8 (10.4)	7 (17.5)	ns
Cancer, n (%)	5 (6.5)	2 (5.0)	ns
Renal, n (%)	2 (2.6)	1 (2.5)	ns
**Symptoms on hospital admission**			
Fever, n (%)	67 (87.0)	16 (40.0)	<0.0001
Cough, n (%)	39 (50.6)	6 (15.0)	0.0001
Shortness of breath, n (%)	23 (29.9)	5 (12.5)	0.0416
Myalgia or arthralgia, n (%)	25 (32.5)	5 (12.5)	0.0249
Diarrhea, n (%)	12 (15.6)	0 (0)	0.0078
Anosmia and ageusia, n (%)	5 (6.5)	0 (0)	ns
Sputum production, n (%)	2 (2.6)	0 (0)	ns
Neurological, n (%)	5 (6.5)	7 (17.5)	ns

n: number, IQR: interquartile range. The 2-tailed X^2 test or Fisher's exact test and the nonparametric comparative Mann-Whitney test were used for comparing proportions and medians, respectively, between accept and decline groups.

Figure S1. (A) Longitudinal evaluation of NfL, GFAP and sCD163 plasma levels according to pulmonary and/or renal comorbidity and (B) according to neurological symptoms presence on hospital admission.

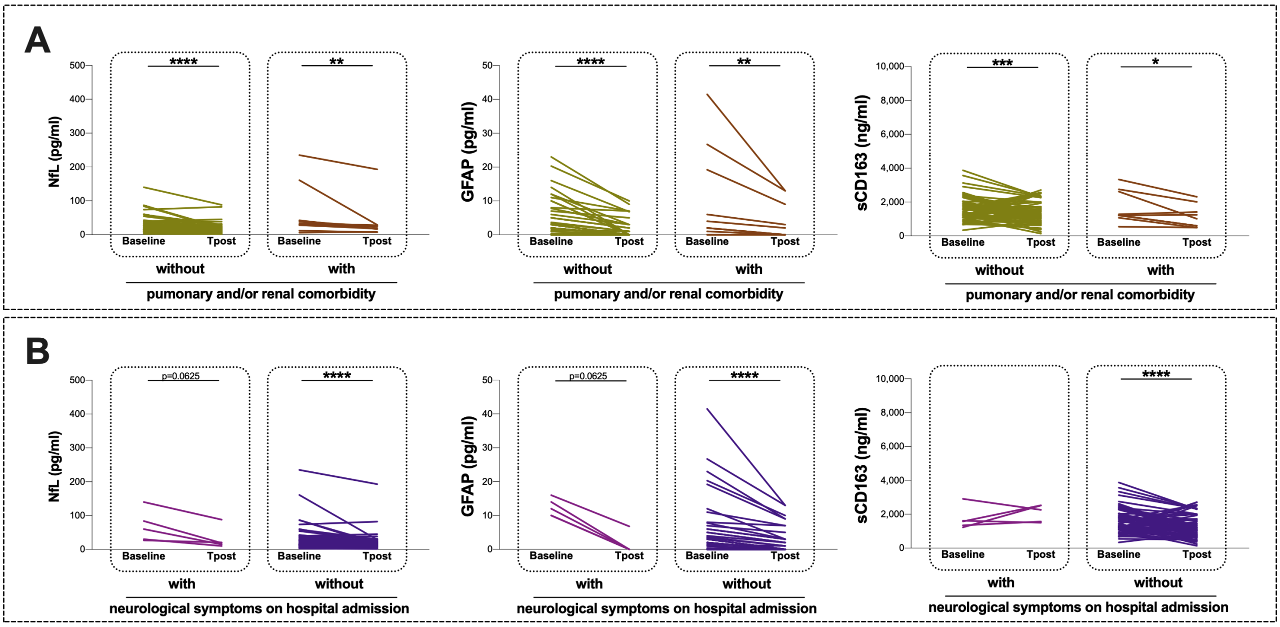

NfL: neurofilament light chain, GFAP: glial fibrillary acidic protein, sCD163: soluble CD163. *: 0.05<p<0.01; **: 0.01<p<0.001; ***: 0.001<p<0.0001; ****: p>0.0001.
